# Kava root extracts hinder prostate cancer development and tumorigenesis by involvement of dual inhibition of MAO-A and LSD1

**DOI:** 10.20517/jtgg.2021.22

**Published:** 2021-05-28

**Authors:** Xuesen Li, Liankun Song, Shan Xu, Matthew Tippin, Shuan Meng, Jun Xie, Edward Uchio, Xiaolin Zi

**Affiliations:** 1 Department of Urology, University of California, Irvine, Orang, CA 92868, USA.; 2 Chao Family Comprehensive Cancer Center, University of California, Orange, CA 92868, USA.; 3 Department of Pharmaceutical Sciences, University of California, Irvine, Irvine, CA 92617, USA.

**Keywords:** Kava, chemoprevention, LSD1, MAO-A

## Abstract

**Aim:**

Here, we aim to evaluate the chemopreventive efficacy of kava root extracts (KRE) in transgenic adenocarcinoma of the mouse prostate (TRAMP) mice and investigate potential molecular targets of kavalactones, the main components of kava.

**Methods:**

TRAMP mice were administrated with KRE formulated food for different periods of time, and then the incidences of high-grade prostatic intraepithelial neoplasia (HG-PIN) and adenocarcinomas and tumor burdens were compared between vehicle control and KRE food fed groups. In addition, the inhibitory effect of the KRE and kavalactones on monoamine oxidase A (MAO-A) and lysine-specific demethylase 1 (LSD1) enzyme activities were examined by commercially available inhibitor screening kits. Histone H3 lysine 9 dimethylation was also evaluated in prostate cancer cells and tumor tissues using Western blotting analysis.

**Results:**

Dietary feeding of 0.3% and 0.6% KRE to TRAMP mice from ages of 6 weeks to 12 weeks inhibited HG-PIN by 43.5% and 59.7%, respectively, and prostate adenocarcinoma by 53.5% and 66.4%, respectively. In addition, 0.6% KRE fed TRAMP mice from ages of 6 weeks to 24 weeks exhibited a significant reduction of genitourinary weight (a surrogate of tumor burden) by 54.5% and reduced body weight gain. Furthermore, the KRE and kavalactones showed a significant inhibition of LSD1 and MAO-A enzyme activities.

**Conclusion:**

Our results suggest that consumption of kava products through diet can delay prostate cancer development and progression and that kavalactones may be a new structure model for developing a potent dual inhibitor of LSD1 and MAO-A.

## INTRODUCTION

Prostate cancer has a projected incidence of ~248,530 new cases diagnosed, accounting for ~34,130 deaths in 2021 in the United States, ranking the second leading cause of cancer death in men^[1]^. The majority of men diagnosed with prostate cancer (about 4 in 5 men) die from other reasons rather than prostate cancer. More than 3.1 million men are alive with a diagnosis of prostate cancer in the United States^[1]^. In addition, over one-third of prostate cancer is slowly growing or progressing over several decades. This group of patients without cancer-related symptoms do not warrant aggressive, immediate treatment^[2]^. Instead, they are closely monitored for their indolent status using prostate-specific antigen (PSA) kinetics, periodic biopsies for histologic progression, and possibly surveillance magnetic resonance imaging (MRI) until further treatment is needed as judged by physicians. This emergent strategy for the management of indolent prostate cancer is called active surveillance^[2]^. Men on active surveillance have high anxiety, depression and doubts about the possible progression of the disease and are highly motivated to prevention approaches using dietary or pharmacological means or positive lifestyle changes^[3]^. Therefore, men on active surveillance have been thought to be an ideal population for chemo/dietary prevention methods to further reduce disease progression and anxiety during the period of active surveillance. Currently very few chemopreventive agents are effective for their testing in chemoprevention studies in this population.

Kava (*Piper methysticum Forst*) is a continually regrowing shrub native to the South Pacific Islands^[4]^. Traditionally, kava root extracts (KRE) have been prepared with coconut milk and water as a drink for a social gathering in the Pacific Islands for thousands of years^[4]^. The kava drink improves sleep quality and produces distinct muscle relaxation without drug addiction^[4]^. Several clinical studies have shown commercially available KRE to reduce anxiety and sold as an anxiolytic agent for several decades^[5–7]^. Kava drinking was also linked to lower cancer risk by an epidemiological study^[8]^. In addition, KRE has demonstrated potent anti-carcinogenic activity in several cancers, including bladder cancer, lung cancer, and others^[9–12]^. Intriguingly, compared to other areas of the world, incidences of prostate cancer in kava drinking countries, such as Fiji, are very low. However, when Fijian men moved to Australia, their prostate cancer incidence raised by 5.1-fold^[13,14]^. These results have led us to investigate whether kava consumption can affect prostate cancer development and progression. We have previously shown that oral administration of KRE through dietary supplementation effectively reduced the growth of patient-derived xenograft (PDX) tumors and down-regulated the protein levels of androgen receptor (AR) and the expression of AR target genes *PSA* and transmembrane protease, serine 2 (*TMPRESS2*)^[9]^. Given the above-described properties of KRE, KRE would have the promise to be further studied as a chemopreventive agent for reducing progression risk and anxiety in prostate cancer patients under active surveillance.

To evaluate the chemopreventive effect of *KRE*, transgenic adenocarcinoma of the mouse prostate (TRAMP) mice were administrated with vehicle control or KRE formulated food in different stages of tumor development to test its ability to prevent prostate cancer development [i.e., occurrence of high-grade prostatic intraepithelial neoplasia (HG-PIN) and prostate adenocarcinomas] or delay progression. In addition, the effects of the KRE and its main components of kavalactones, including kawain, dihydrokawain, yangonin, and methysticin, on monoamine oxidase A (MAO-A) and lysine-specific demethylase 1 (LSD1) enzyme activities were examined. Our results have shown that the KRE significantly reduced the occurrences of HG-PIN and prostate adenocarcinomas and slowed tumor growth in the TRAMP transgenic mice, and inhibited both MAO-A and LSD1 activities in prostate cancer cells.

## METHODS

### Study materials

Authenticated LNCaP and C4–2B cell lines without mycoplasma contamination were used as described in details in our previous publication^[15]^. The KRE (150 mg/mL kavalactones in 50% ethanol) was purchased from Gaia Herbs (Brevard, NC). Main kavalactones, including kawain, 5’,6’-dehydrokawain, yangonin, and methysticin, were isolated and purified by LKT Laboratories, Inc. (St. Paul, MN) from the KRE. Antibodies against MAO-A and tubulin were from Santa Cruz Biotechnology, Inc. (Santa Cruz, CA). Di-methylated histone H3 lysine 4 (H3K4) and H3K9 and Ki67 antibodies were purchased from Abcam (Cambridge, MA). MAO-A and LSD1 activity assay kits were from Cayman Chemical Company (Ann Arbor, Michigan).

### Prevention and intervention protocols in TRAMP mice

A cohort of male hemizygous TRAMP mice was obtained by breeding female hemizygous C57BL/TGN TRAMP mice with male FVB/N mice and genotyped by a polymerase chain reaction (PCR) method as described in details in our previous publication^[16]^. The 0.3% (3 g/kg food) or 0.6% (6 g/kg food) KRE containing rodent food was formulated into AIN-93M rodent food by Dyets, Inc. through customer services. University of California, Irvine approved protocol (#2007–2740) was followed for animal care and treatments. For the prevention protocol, 0.3% or 0.6% KRE containing food or vehicle control food was given to TRAMP mice from ages of 6 weeks to 12 weeks. For the intervention protocol, 0.3% or 0.6% KRE containing food or vehicle control food was administrated to 6 weeks old TRAMP mice until they were at the age of 24 weeks old.

The body weight and food consumption were recorded weekly until the end of the experiments. Organ weights, including liver, spleen, kidney, *etc.* and genitourinary (GU) weights were also measured at the end of the experiments and fixed in formalin for standard H&E slide preparation and examination^[16]^. PIN lesions and prostate adenocarcinoma were evaluated to Dr. Cardiff’s description^[17]^ and our previous publication^[16]^.

### LSD1 inhibition assay^[18]^

LSD1 inhibitor screening kit was purchased from Cayman Chemicals with the catalog number 700120. Human recombinant LSD1 enzymes are incubated with a selected concentration of compounds or with DMSO for 10 min. Then a premix of reaction buffers solution that contains fluorescence substrate and horseradish peroxide (HRP) was added to the LSD1 solution. Finally, methylated peptides, which are the first 21 amino acids of the N-terminal tail of methylated H3K4, were added to the LSD1-buffer solution to begin the enzymatic processing. This process took place at 37 °C for 60 min. In the process of demethylation, hydrogen peroxide (H_2_O_2_) is formed as a byproduct. HRP then can use H_2_O_2_ to convert the non-fluorescence substrate resazurin into the fluorescence substrate resorufin, which was measured by a fluorescence spectrophotometer using the excitation value of 530 nm and the emission value of 585 nm.

### MAO-A inhibition assay^[19–21]^

MAO-A activity assay kit was purchased from Cayman Chemicals. The measurement of MAO-A activity in vehicle control (0.1% DMSO) or kavalactones (50 μM) or the KRE (5 μg/mL) treated LNCaP and C4–2B cells were carried out by following the kit instruction. HRP used the H_2_O_2_ byproduct in the MAO-A reaction to convert the non-fluorescence substrate resazurin into the fluorescence substrate resorufin, which was measured by a fluorescence spectrophotometer using the excitation value of 530 nm and the emission value of 585 nm.

### Western blot analysis

Protein lysates preparation and Western blotting analysis were performed according to our previously published paper^[22]^. Tubulin levels were used as a loading control.

### Immunohistochemistry

Antigen retrieval method, titration of anti-human Ki67 (1:100) antibody, and immunohistochemistry staining of prostate tissues from control and the KRE containing food fed TRMAP mice were carried out by following our published papers^[9,16]^.

### Molecular docking studies^[23]^

First, kawain and methysticin molecules were generated by Pymol software and converted to the pdbqt format using Open Babel. Then, the autodock 4 software from the Scripps Research Institute was used to dock kawain and methysticin with LSD1 protein (PDB code: pdb 2hkO) or MAO-A (pdb 2bxs) from MGLTools. Finally, Pymol was used to image the conformations of the molecule and protein interaction.

### Statistical analysis

Analysis of variance or Student’s *t*-test followed by the Bonferroni *t*-test for multiple comparisons was used to compared means of organ and body weights and food consumptions between vehicle control and KRE treatments over time. The Mann-Whitney *U* and Kolmogorov-Smirnov test was used for GU weight comparison among different treatment groups. The comparisons of the percentages of mice with different pathologic stages or with palpable tumors among different treatment groups were tested by using the *χ*^2^ or Fisher exact test.

## RESULTS

### Dietary feeding of the KRE for 6 weeks inhibits mouse HG-PIN and prostate adenocarcinoma in TRAMP mice

**Figure 1.**
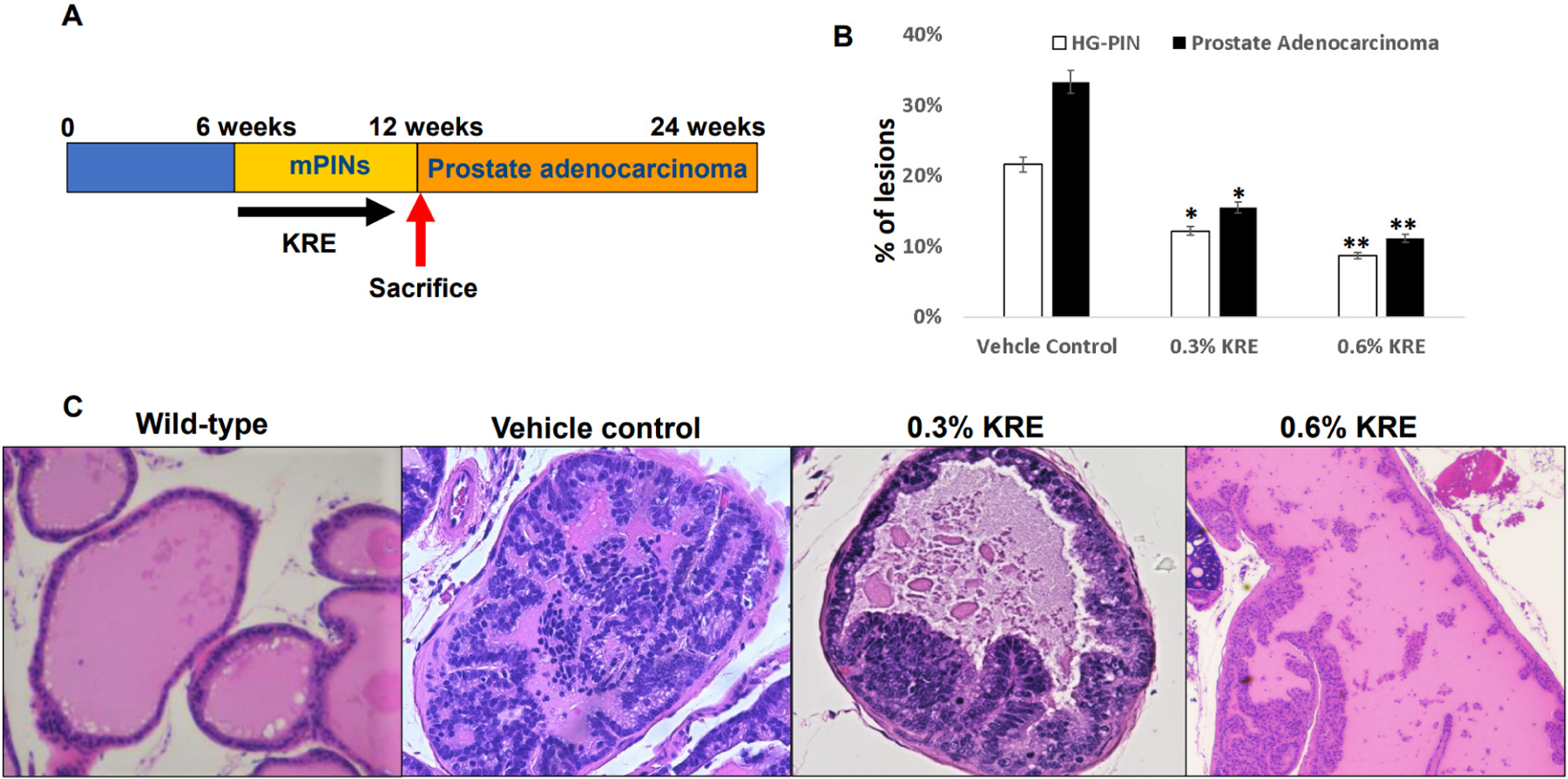
Dietary feeding of the KRE inhibits the occurence of HG-PIN and prostate adenocarcinoma in TRAMP mice. (A) A simple illustration of prevention protocol by the KRE in the TRAMP model. (B) Percentages of HG-PIN or adenocarcinoma lesions in the prostates of 12-week-old, vehicle control *vs.* 0.3% or 0.6% KRE conatining food administrated to TRAMP mice. The prostates were from 8 mice in each group, and the total number of prostate acinus was counted. Fisher exact test show *P* < 0.05. (C) Prostates in 12-weeks-old, vehicle control *vs.* 0.3% or 0.6% KRE containing food fed TRAMP mice were examined by H&E staining and histological evaluation. 200× magnifications of images are shown. KRE: Kava root extract; HG-PIN: high-grade prostatic intraepithelial neoplasia; TRAMP: transgenic adenocarcinoma of the mouse prostate. *P < 0.05 and **P < 0.01, respectively.

To examine whether the KRE inhibits HG-PIN and prostate adenocarcinoma occurrence, TRAMP mice were given vehicle control, 0.3% KRE, or 0.6% KRE containing food from 6 weeks of age to 12 weeks of age [Figure 1A]. Figure 1B and C show that dietary feeding of 0.3% and 0.6% KRE inhibited HG-PIN by 43.5% and 59.7%, respectively, and prostate adenocarcinomas by 53.5% and 66.4%, respectively (*P* < 0.05; Fisher exact test). These results suggest that the KRE has a cancer-preventive activity for early-stage prostate cancer.

### Dietary feeding of the KRE for 18 weeks reduced tumor burden in TRAMP mice

**Figure 2.**
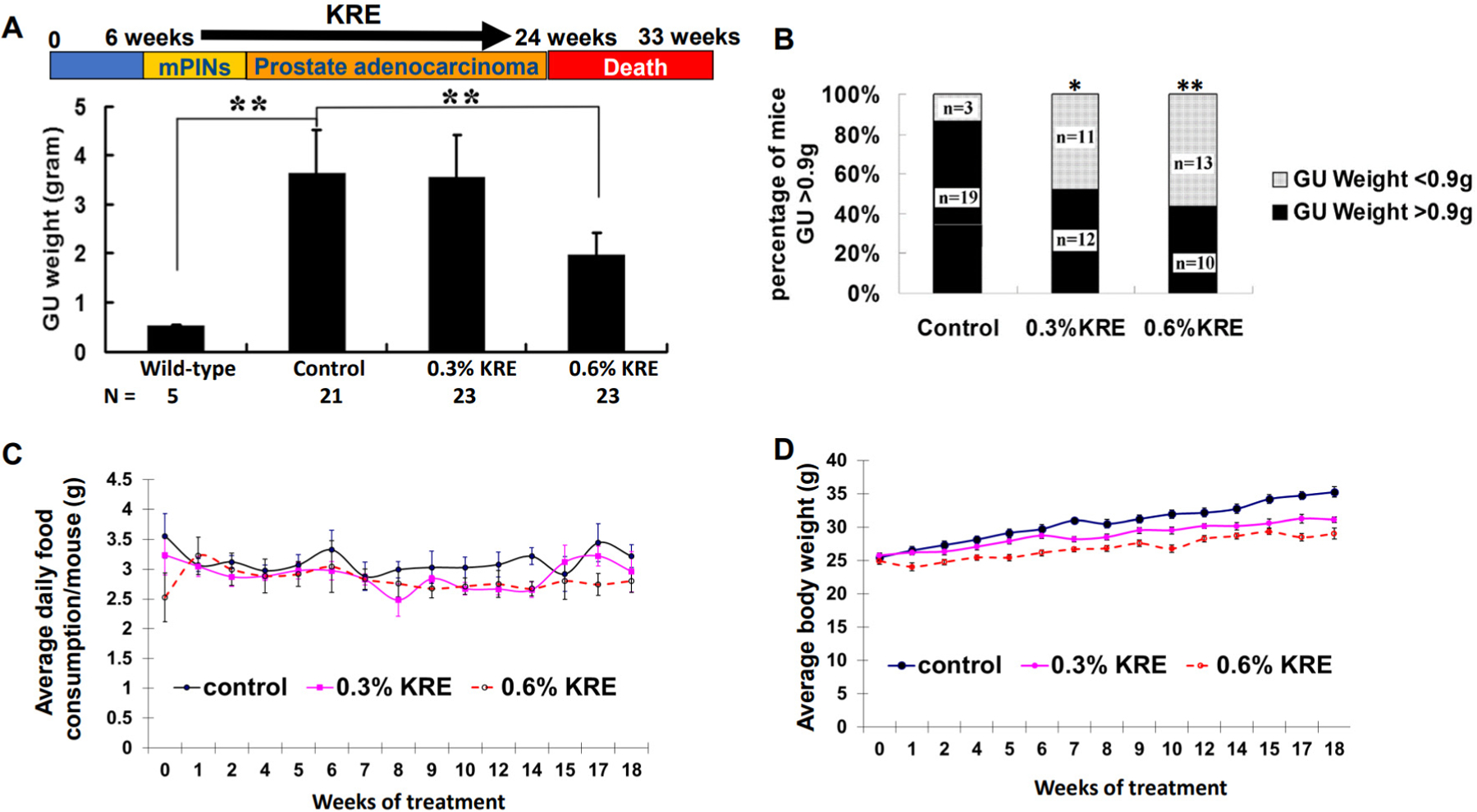
Dietary feeding of the KRE reduces tumorigenesis in TRAMP mice. (A) Upper panel, schematic presentation of intervention protocol by the KRE in the TRAMP model. Lower panel, average GU weights in different treatment groups. **P < 0.01. (B) Percentages of mice with large tumor formation. *P < 0.05 and **P < 0.01. (C) 0.6% KRE food results in a reduction of food consumption over time. (D) Dietary feeding of 0.3% and 0.6% KRE food reduces mean body weight gain of TRAMP mice over time. KRE: Kava root extract; TRAMP: transgenic adenocarcinoma of the mouse prostate; GU: genitourinary.

To determine whether the KRE can affect tumor burdens at late stage of prostate cancer, TRAMP mice at 6 weeks of age were administrated with vehicle control, 0.3% or 0.6% KRE containing food for 18 weeks. The mean GU weight of the mice fed with 0.6% KRE containing food was significantly reduced when compared to the control group (Figure 2A; 1.98 g ± 2.07 g *vs.* 3.63 g ± 4.28 g; *P* < 0.05; Mann-Whitney *U* and Kolmogorov-Smirnov test). The percentages of large tumors (GU weight > 0.9 g) were also decreased from 86.4% in the control group to 52.2% and 43.5% in 0.3% and 0.6% KRE food groups, respectively [Figure 2B]. In addition, administration of 0.6 % KRE containing food to TRAMP mice led to a decrease in food consumption, and both 0.3% and 0.6% KRE food suppressed the body weight gain over time [Figure 2C and D], as well as increased liver weight (data not shown) compared to control food.

### Kavalactones and KRE inhibit MAO-A activities in prostate cancer cell lines LNCaP and C4–2B cells

**Figure 3.**
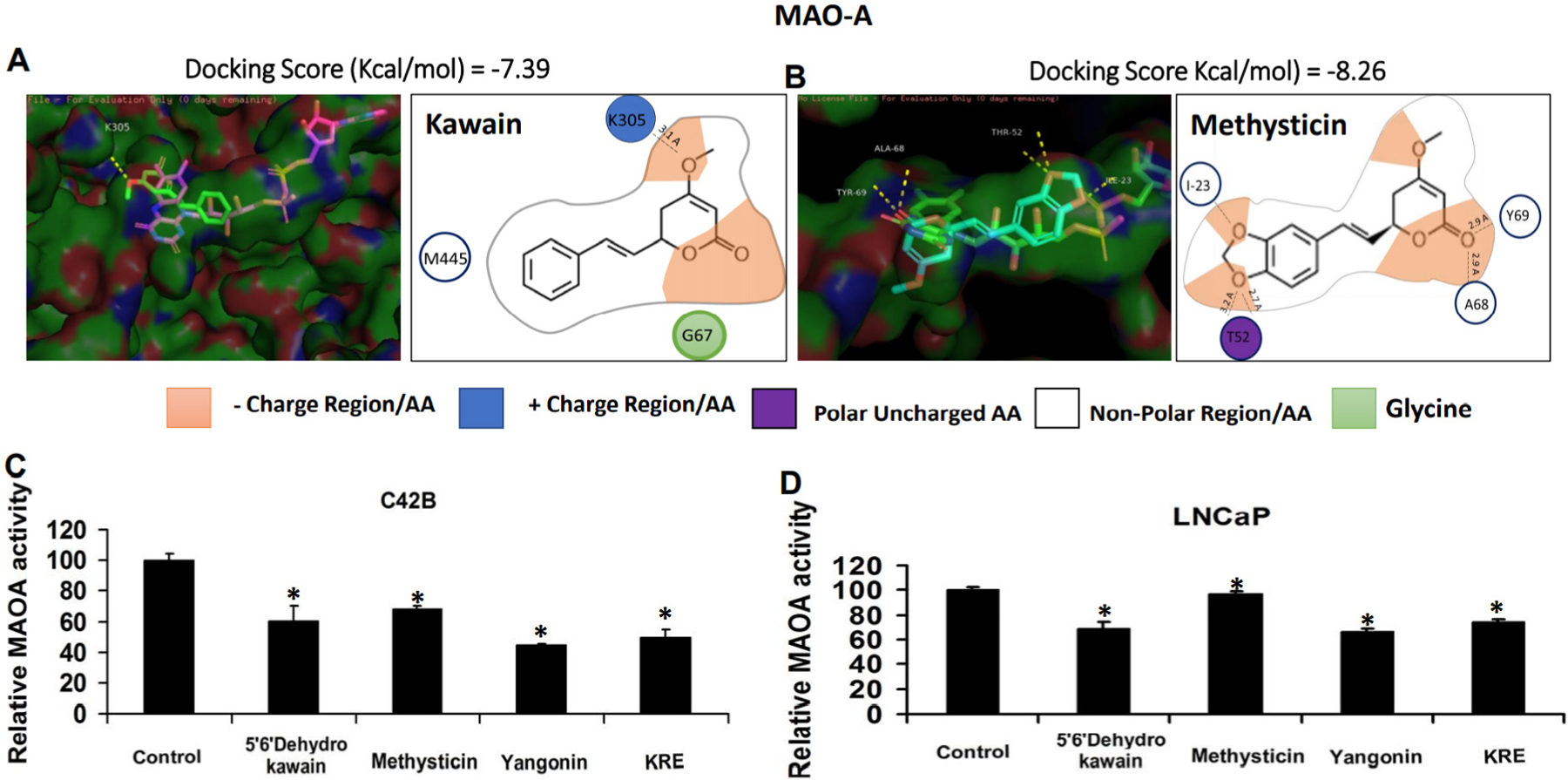
Kavalactones inhibits MAO-A enzyme activity. (A, B) Kawain and methysticin were docked with MAO-A protein by Autodocktools program, and the superimposed binding of kawain and methysticin to MAO-A, respectively, was observed in Pymol program. 2D molecular structures of kawain and methysticin are shown to interact with key amino acids on the predicted binding site. (C) MAO-A enzyme activities were analyz ed in prostate cancer C4–2B and LNCaP cell lysates after treatment with 0.1% DMSO (control), KRE and kavalactones for 24 h. MAO-A: Monoamine oxidase A; KRE: kava root extract. *P < 0.05.

Several studies reported that MAO-A was associated with aggressive prostate cancer and promoted prostate tumor growth and metastasis^[20,21,24]^. In addition, MAO-A is a key target for the treatment of neuropsychiatric and neurodegenerative disorders^[19]^. We therefore have examined whether the KRE and its active components kavalacotones can inhibit cellular MAO-A activity in LNCaP and C4–2B cells. First, we used the autodock program to dock kavalactones with MAO-A protein (PDB code: pdb 2bxs). Kawain and methysticin have predicted IC_50_ values of 3.85 μM and 880.78 nM, respectively. Pymol program was used to observe the superimposed binding of kawain and methysticin to the flavin adenosine dinucleotide (FAD)-binding and the active site of MAO-A and the kavalactones were predicted to form hydrogen bonds [Figure 3A and B].

In addition, the cellular MAO activities were examined by MAO-GLO assay after LNCaP and C4–2B cells were treated with KRE and kavalactones for 24 h. Figure 3C and D show that kavalactones and KRE significantly inhibited cellular MAO activities by about 25% to 51% (*P* < 0.05). These results suggest that kavalactones and KRE are weak inhibitors of MAO activity in prostate cancer cells.

### Kavalactones inhibit LSD1 activities in prostate cancer cells and dietary feeding of KRE is associated with reduced LSD1 activities in prostate tumors

**Figure 4.**
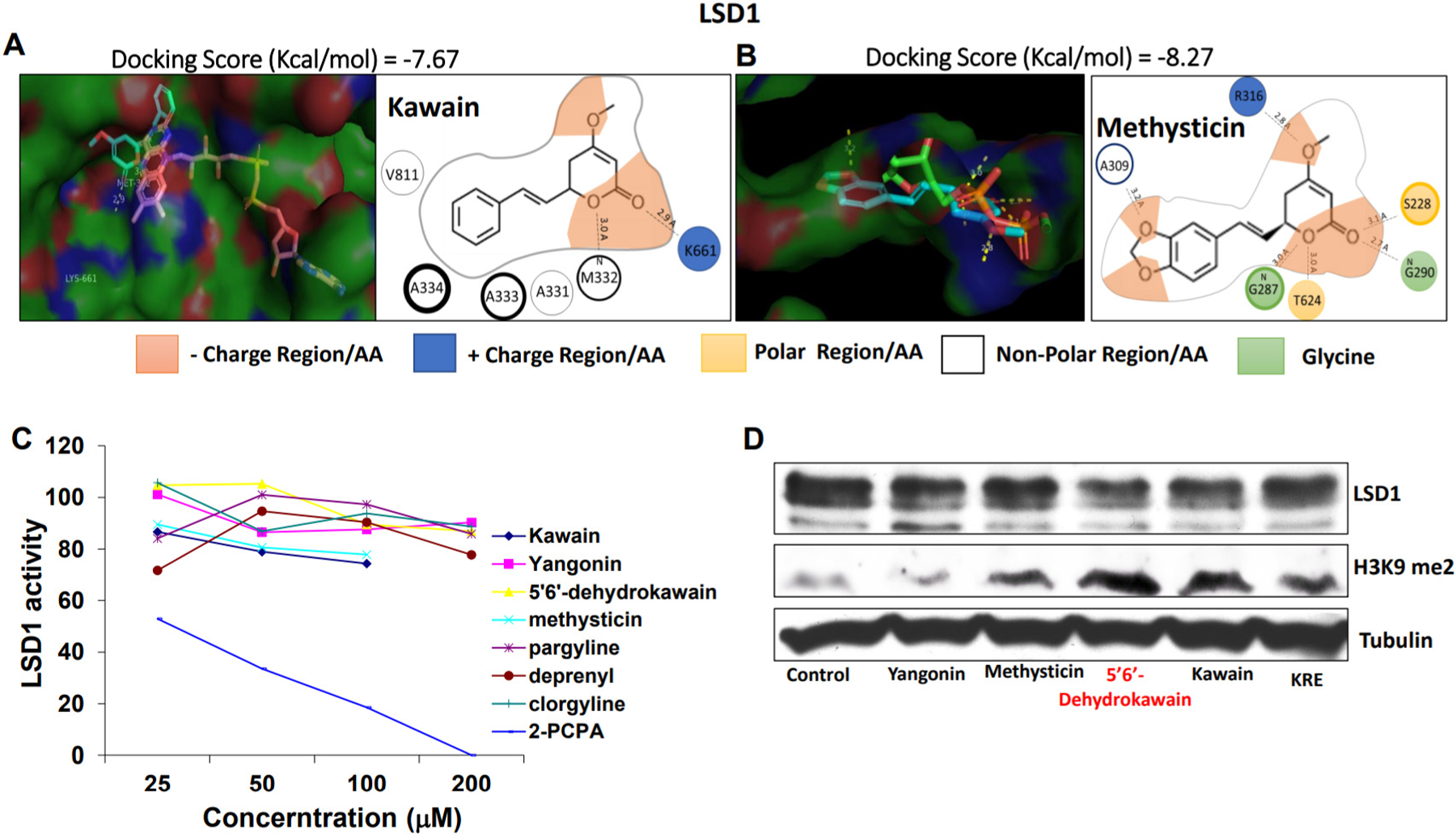
Kavalactones inhibits LSD1 enzyme activity. (A, B) Autodocktools program was used to dock kawain and methysticin with LSD1 protein, and Pymol program was used to observe the superimposed binding of kawain and methysticin to LSD1, respectively. 2D molecular structures of kawain and methysticin are shown to interact with key amino acids within the predicted binding site. (C) *In vitro* LSD1 enzyme activities were analyzed using LSD1 inhibitor screening kit. (D) Western blotting analysis of LSD1 expression and H3K9 dimethylation. LSD1: Lysine-specific demethylase 1.

LSD1 is an epigenetic enzyme responsible for demethylation of H3K4 and H3K9 for reprogramming gene expression profile and suggested to have a critical role in the progression of castration-resistant prostate cancer via interacting with AR^[25,26]^. Given the catalytic activity of both MAO-A and LSD1 resides in the amine oxidase domain and is dependent on its co-factor FAD^[18]^, we therefore examined whether kavalactones could also affect LSD1 activity in prostate cancer. Molecular docking analysis revealed that kawain and methysticin superimpose FAD and binds to the FAD-binding domain of LSD1 and interacts with important amino acids, methionine 332 and arginine 316, respectively [Figure 4A and B].

*In vitro* LSD1 inhibitory activity assay shows that kawain and methysticin have the most significant inhibition against LSD1 enzyme activity among the tested kavalactones [Figure 4C]. In addition, protein expression levels of dimethylated H3K9 were observed to be significantly increased in KRE, methysticin, 5’,6’-dehydrokawain, or kawain treated LNCaP cells without affecting LSD1 expression [Figure 4D]. Our previous studies have also reported that KRE, methysticin and kawain inhibited the gene transcription of *PSA* and *TMPRSS2*, two AR target genes. These results together suggest that KRE, methysticin, and kawain reduce LSD1 enzyme activity in cells leading to inhibition of AR signaling.

### KRE containing food increased H3K9 dimethylation and reduced Ki67 expression in tumor tissues

**Figure 5.**
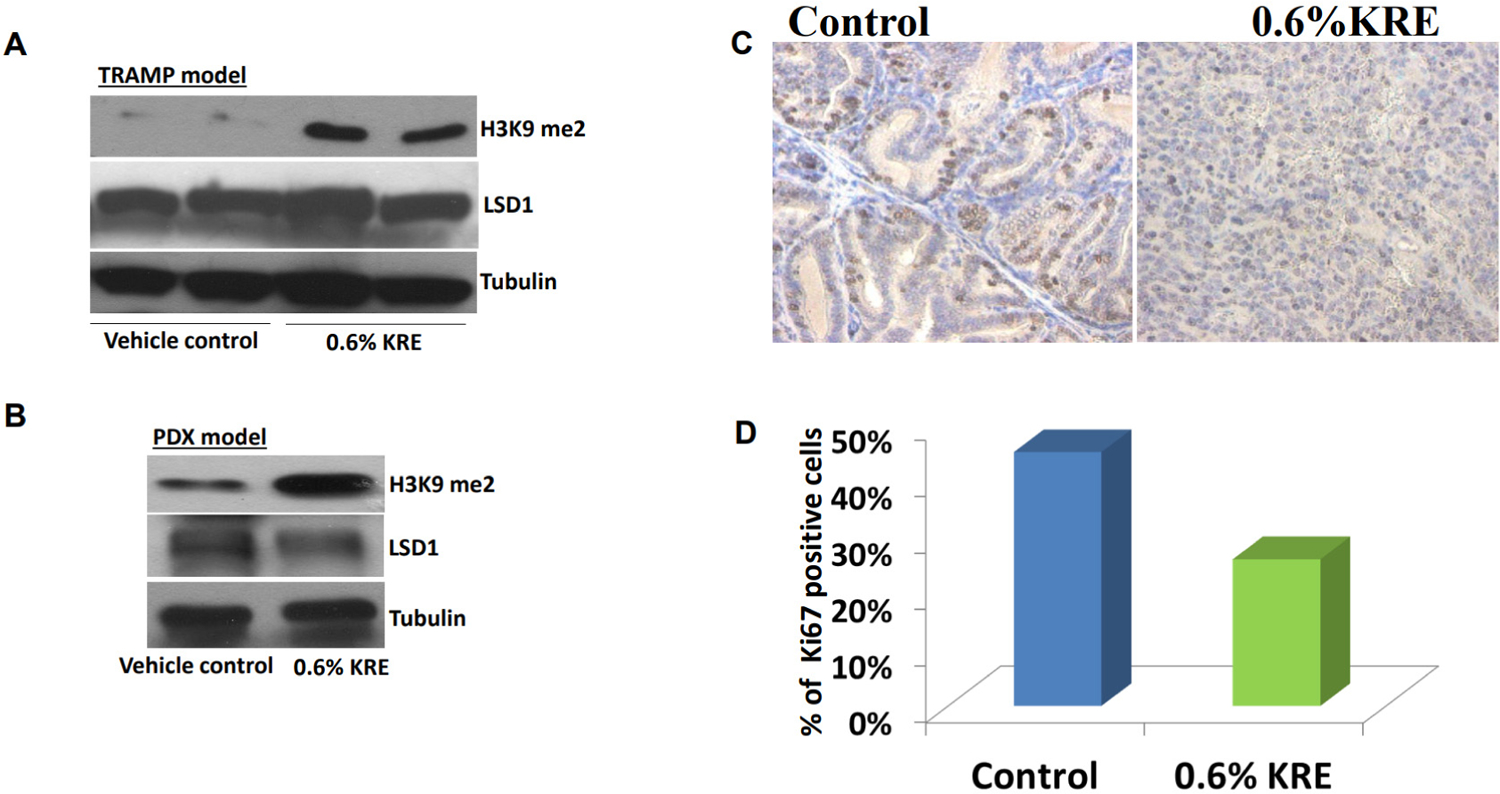
Dietary feeding of KRE enhances H3K9 dimethylation and inhibits cell proliferation *in vivo*. (A, B) Western blot analysis of H3K9 dimethylation in mouse dorsolateral prostates of TRAMP mice and prostate cancer PDX tumors, respectively, in vehicle control and 0.6% KRE treatment groups. (C) Ki67 immunohistochemistry staining was shown in the prostates of TRAMP mice that was treated with vehicle control *vs.* 0.6% KRE containing food for 18 weeks. 200× magnifications of images are presented. (D) The percentage of positive stained cells was calculated after 20 fields from ten mouse prostates were counted in each group. KRE: Kava root extract; H3K9: histone H3 lysine 9; TRAMP: transgenic adenocarcinoma of the mouse prostate; PDX: patient-derived xenograft; LSD1: lysine-specific demethylase 1.

Similar to the *in vitro* findings as described above, Western blotting analysis confirmed that KRE containing food increased H3K9 dimethylation without obvious effects on LSD1 expression in prostate tissues of the TRAMP mice [Figure 5A] and tumor tissues of a prostate cancer PDX model from our previous studies [Figure 5B]. In addition, immunohistochemistry analysis revealed that the number of Ki67-positive cells in the prostate of KRE-fed TRAMP mice was 26.3% ± 4.7% compared with 49.1% ± 10.2% in those from the vehicle control treatment (*P* < 0.05; Figure 5C and D). This finding suggests that KRE feeding inhibited LSD1 activity, leading to the anti-proliferative effects of KRE on prostate tumor tissues of mouse models and therefore delaying the progression of prostate cancer.

## DISCUSSION

Although kavalactones have been suggested to be responsible at least in part for both the anxiolytic effect and the anti-prostate cancer effect, the molecular targets of kavalactones remain understudied. In this study, our results have shown that kawain and methysticin are weeks but cell-active inhibitors for both MAO-A and LSD1 enzymes. Therefore, our study has provided the first structure base for further developing more potent dual inhibitors of MAO-A and LSD1 through chemical modification of the kavalactone skeleton. Given both MAO-A and LSD1 have been demonstrated to be critically involved in prostate cancer progression. Dual inhibition of MAO-A and LSD1 may have significant translation values in prostate cancer prevention and treatment.

LSD1 was reported to interact with the AR and serves as a co-factor to regulate the transcriptional expression of AR target genes^[25,26]^. Our previous studies demonstrated that kavalactones inhibited mRNA expression of *PSA* and *TMPRESS2*, two key AR target genes, in LNCaP cells and tumor tissues of prostate cancer PDX model^[9]^. Whether the inhibitory effect of kawain and methysticin on AR signaling is dependent, at least in part, on LSD1 expression warrants further studies. These studies will facilitate further understanding of molecular mechanisms by which these kavalactones regulate AR transcription.

Previous studies by our group and others have demonstrated that kava chalcones (flavokawain A) and a kavalactone-rich kava fraction B (free of flavokawains), respectively, are effective in preventing the occurrences of HG-PIN and early prostate adenocarcinoma and reducing tumor burdens in the TRAMP model^[10,16]^. In addition, we have shown that administration of flavokawain A containing food to TRAMP mice resulted in the eradication of distant organ metastasis^[16]^, whereas a kavalactone-rich kava fraction B was shown to significantly decrease the incidences of neuroendocrine carcinoma in TRAMP mice by absolute 42.9%^[10]^. The KRE used here contains both kavalactones and flavokawains, including 2.7% kawain, 1.4% methysticin, 1.75% 5,6-dehydrokawain, 3.08% Yangonin, 0.33% flavokawain B, and 0.21% flavokawain A^[9]^. The KRE may be able to target different stages and heterogenity of prostate cancer. Consistent with the previous studies, the KRE has similar potency as a kavalactone-rich kava fraction B for its chemopreventive effect in prostate cancer in the TRAMP model. However, further studies are still needed to investigate whether there are significant synergistic or antagonistic effects between flavokawains and kavalactones on prostate cancer.

Dietary feeding of the KRE used here resulted in decreased body weight gain and liver enlargement (data not shown). This result is also consistent with the report by Tang *et al.*^[10]^ that the kavalactone-rich kava fraction B (free of flavokawains) suppressed body weight gain and induced liver enlargement (hepatomegaly) without noticeable changes in liver function. In contrast, the changes in body weight gain and organ weight after long-term dietary feeding of flavokawain A in TRAMP mice have not been observed in our previous studies^[16]^. Therefore, it is unlikely that flavokawain A is responsible for the reported “kava hepatotoxicity”. Flavokawain A could be a safer product for cancer prevention than the KRE and the kavalactone-rich kava fraction B.

In summary, the KRE product containing both kavalactones and flavokawains reduced the incidence of HG-PIN and early-stage prostate adenocarcinoma and inhibited tumorigenesis. The KRE and kavalactone weakly inhibited both MAO-A and LSD1 activities in prostate cancer, leading to their inhibitory effect on AR signaling and cell proliferation. This study has provided a new knowledge basis for developing more effective dual inhibitors of MAO-A and LSD1 enzymes for prostate cancer prevention and treatment.
